# Emerging genetic diversity and molecular epidemiology of *Entamoeba moshkovskii* among patients with acute diarrhoea in Northern India

**DOI:** 10.1186/s41182-025-00876-5

**Published:** 2026-01-03

**Authors:** Puja Garg, Pankaj Malhotra, Surinder Singh Rana, Sadhna Lal Bhasin, Rakesh Sehgal, Priya Datta

**Affiliations:** 1 Department of Medical Parasitology, PGIMER, Chandigarh, 160012 India; 2 Department of Clinical Haematology and Oncology, PGIMER, Chandigarh, 160012 India; 3 Department of Gastroenterology, PGIMER, Chandigarh, 160012 India; 4 Department of Paediatrics Gastroenterology, PGIMER, Chandigarh, 160012 India; 5 Department of Microbiology, Aarupudai Veedu Medical College & Hospital, VMRF-DU, Puducherry, India

**Keywords:** *Entamoeba moshkovskii*, Acute diarrhoea, Real-time PCR, Genetic diversity, Phylogenetic analysis, Zoonotic transmission, Diagnostics, One Health

## Abstract

**Background:**

*Entamoeba moshkovskii*, a morphologically indistinguishable but genetically distinct species from *E. histolytica*, has recently emerged as a potential cause of human diarrhoeal disease. Despite its increasing global recognition, its epidemiological role, genetic diversity, and transmission dynamics remain poorly defined, particularly in the Indian subcontinent.

**Methods:**

A prospective study was conducted among 300 hospitalised patients with acute diarrhoea in Northern India to determine the molecular prevalence and phylogenetic diversity of *E. moshkovskii*. Stool samples were examined microscopically and tested using a species-specific real-time polymerase chain reaction (PCR) assay targeting the 18S rRNA gene. Positive amplicons were sequenced bidirectionally and compared with global reference sequences to infer genetic relationships and lineage divergence.

**Results:**

*E. moshkovskii* DNA was detected in 17 of 300 patients (5.7%; 95% confidence interval 3.3–8.9%), with higher detection among male and paediatric participants. Twelve isolates yielded high-quality sequences showing 99–100% identity with reference strains, while three exhibited a reproducible thymine-to-purine substitution at position 1655. Phylogenetic reconstruction revealed three major clusters—human, animal, and environmental—with most isolates forming a human-associated lineage and two clustering with non-human strains, suggesting cross-ecological transmission.

**Conclusions:**

This study provides the first sequence-confirmed evidence of *E. moshkovskii* infection in Northern India and demonstrates emerging genetic diversity among clinical isolates. The coexistence of clonal and divergent strains highlights complex transmission pathways involving environmental and zoonotic reservoirs. These findings underscore the need to incorporate *E. moshkovskii* into molecular diagnostic algorithms and diarrhoeal disease surveillance within a One Health framework.

**Supplementary Information:**

The online version contains supplementary material available at 10.1186/s41182-025-00876-5.

## Introduction

Amoebiasis, caused primarily by the intestinal protozoan *Entamoeba histolytica*, remains a major parasitic infection of global health importance. It affects approximately 50 million individuals and results in nearly 100,000 deaths annually, predominantly in low- and middle-income countries where sanitation and hygiene are compromised [1, 2]. Transmission occurs through the faecal–oral route via ingestion of cysts, which release trophozoites in the intestine. While most infections remain asymptomatic, invasive disease can manifest as amoebic colitis and, in severe cases, extraintestinal involvement such as amoebic liver abscess [3]. A complex interplay of parasite virulence, host immunity, and environmental exposure therefore shapes the clinical spectrum of amoebiasis.

The genus *Entamoeba* comprises several morphologically indistinguishable species, including *E. histolytica*, *E. dispar*, *E. bangladeshi*, and *E. moshkovskii* [4]. Of these, *E. histolytica* is the only species unequivocally established as pathogenic, whereas *E. dispar* and *E. bangladeshi*, first described from Bangladesh, have subsequently been detected in both asymptomatic and symptomatic individuals from Bangladesh, India, and South Africa; however, their pathogenic potential remains unconfirmed and requires further investigation [5–7]. The pathogenic potential of *E. moshkovskii*, however, remains unresolved [8, 9]. Earlier considered a free-living amoeba, *E. moshkovskii* was primarily associated with saline and sewage environments. Subsequent molecular investigations, however, have demonstrated its presence in human, animal, and environmental samples across diverse geographic regions, including India, Bangladesh, Malaysia, Iran, Australia, Colombia, and Tanzania [10–13]. Its occurrence in treated wastewater and agricultural reuse systems underscores its environmental resilience and potential for waterborne transmission [14], while detection in animal hosts such as pigs in Eastern India suggests possible zoonotic routes of infection [15].

Although *E. moshkovskii* shares morphological features with *E. histolytica*, their clinical outcomes appear heterogeneous. In a Bangladeshi cohort, *E. moshkovskii* was the sole pathogen detected in children with acute diarrhoea [12], and in experimental murine models, infection produced diarrhoea, colitis, and weight loss [12]. Conversely, its detection in asymptomatic individuals, including HIV-positive patients in Tanzania, highlights the influence of host immunity and environmental factors on disease manifestation [16, 17]. These contrasting observations underscore the need to delineate the biological and pathogenic variability of *E. moshkovskii*.

Microscopic identification alone cannot reliably distinguish *E. moshkovskii* from *E. histolytica*, *E. dispar*, or *E. bangladeshi,* leading to substantial diagnostic ambiguity and misclassification [18, 19]. This limitation has been addressed by molecular assays targeting conserved genomic regions such as the 18S rRNA gene, which enable species-specific differentiation and epidemiological tracking [20].

Several molecular studies have reported substantial genotypic variation within *E. moshkovskii* populations, including distinct 18S rRNA sequence variants, novel genotypes, and single-nucleotide polymorphisms (SNPs), indicating greater diversity than previously recognised. Molecular approaches, including PCR and sequencing-based analyses, have further revealed the presence of genetically distinct clades potentially adapted to human, animal, and environmental niches, suggesting ongoing diversification within this species complex. However, the epidemiology, genetic diversity, and clinical relevance of *E. moshkovskii* remain poorly characterised in India, particularly in northern regions where molecular surveillance is scarce.

Although *E. moshkovskii* has been increasingly reported from Eastern and Southern India, molecular and sequence-based data from Northern India remain virtually absent. Only one prior cross-sectional study from this region examined multiple Entamoeba species collectively, without sequence confirmation or phylogenetic analysis of *E. moshkovskii* [21]. In contrast, the present study focuses exclusively on sequence-verified detection and genetic characterisation of *E. moshkovskii* among hospitalised patients with acute diarrhoea.

In view of these diagnostic and epidemiological uncertainties, the present study aimed to (i) determine the molecular prevalence of *E. moshkovskii* among patients with acute diarrhoea in hospitalised patients of a tertiary care centre in Northern India; (ii) perform phylogenetic analysis to assess genetic relatedness between clinical isolates and reference strains from human, animal, and environmental sources; and (iii) identify single-nucleotide polymorphisms (SNPs) or deletions through mutation analysis to explore evolutionary divergence and diagnostic implications. All *E. moshkovskii*-positive samples will be screened for co-infections, and only symptomatic hospitalised cases will be included to establish regional molecular evidence. By integrating molecular detection, phylogenetic reconstruction, and mutational profiling, this study aims to generate new insights into the epidemiology and genetic variability of *E. moshkovskii*, thereby contributing to a clearer understanding of its role in human disease within a One Health framework.

## Materials and methods

### Study design and ethical approval

This prospective, observational study was conducted between January 2023 and January 2024 at the Postgraduate Institute of Medical Education and Research (PGIMER), Chandigarh, India. The study protocol was reviewed and approved by the Institutional Ethics Committee (IEC/INT/2022/SPL-1243; dated 6 October 2022). Written informed consent was obtained from all adult participants, and from parents or legal guardians for minors, in accordance with the Declaration of Helsinki.

### Study population

Hospitalised patients with acute diarrhoea admitted to the Departments of Paediatric Gastroenterology, Gastroenterology, and Clinical Haematology & Medical Oncology were prospectively enrolled. Acute diarrhoea was defined as the passage of three or more loose or watery stools within 24 h, with symptom duration of less than 14 days (WHO). Patients receiving antiparasitic therapy within the preceding month or with incomplete clinical information were excluded. All samples were obtained from hospitalised patients who underwent routine stool microscopy as part of standard clinical evaluation. No bacterial culture or viral antigen testing was performed within the scope of this study.

### Sample collection and transport

From each participant, three stool samples were collected on three consecutive days in clean, wide-mouthed, leak-proof containers labelled with unique patient identifiers, to improve diagnostic sensitivity in accordance with standard parasitological guidelines. Samples were transported under cold-chain conditions (4 °C) to the Department of Medical Parasitology and processed within two hours of collection. When immediate processing was not feasible, aliquots were stored at − 20 °C for up to 48 h before DNA extraction. A patient was considered positive if any one of the three samples tested positive by microscopy and/or PCR, following the CDC recommendations for intestinal parasite diagnosis [22]

### Microscopic examination

Each stool specimen was screened microscopically for intestinal parasites. Direct wet mounts were prepared in normal saline and Lugol’s iodine and examined for trophozoites and cysts under 10 × and 40 × magnifications using a bright-field microscope. Stool concentration was performed using the formalin–ether sedimentation method to enhance detection sensitivity. Also, Trichrome stain was done following the protocol provided by Datta et al. [23], and microscopy results were recorded for all samples before proceeding to molecular testing.

### Genomic DNA extraction

Genomic DNA was extracted from approximately 200 mg of each stool specimen using the QIAamp Fast DNA Stool Mini Kit (QIAGEN, USA), following the manufacturer’s instructions. Extractions were performed under aseptic conditions to minimise cross-contamination. DNA was eluted in 100 µL of elution buffer and stored at − 20 °C until further use. The concentration and purity of extracted DNA were assessed using a NanoDrop 2000 spectrophotometer (Thermo Fisher Scientific, USA). Samples exhibiting A260/280 ratios between 1.8 and 2.0 were considered suitable for molecular diagnosis.

### Real-time PCR assay for *Entamoeba moshkovskii*

Detection of *E. moshkovskii* was performed using a previously published probe-based real-time PCR assay targeting the 18S rRNA gene, as described by Hamzah et al. [27]. The assay amplifies a ~128-bp fragment within the 18S rRNA region containing diagnostic polymorphic sites that distinguish *E. moshkovskii* from *E. histolytica* and *E. dispar* [18]. In addition, the real-time PCR panel incorporated species-specific primers and probes for *E. histolytica* and *E. dispar*, as previously reported by Ali and Roy [24], to ensure comprehensive detection and differentiation among the human-infecting *Entamoeba* species.

Amplification reactions (15 µL total volume) contained 7.5 µL of master mix, 0.5 µL each of forward and reverse primers, 0.5 µL of probe (5′–FAM–TGT GAA TGG CAA TGG C–BHQ1–3′), 2 µL of DNA template, and 4 µL of nuclease-free water. Reactions were run on a Bio-Rad real-time PCR system with the following thermal cycling profile: initial denaturation at 95 °C for 5 min, followed by 35 cycles of 95 °C for 10 s and 50 °C for 10 s.

For assay validation and as a positive control, reference DNA was derived from a previously confirmed *E. moshkovskii*-positive clinical sample obtained from the same study cohort. The identity of this reference sample was verified by Sanger sequencing of the 18S rRNA gene, which showed 100% sequence identity with published *E. moshkovskii* reference sequences (GenBank accession KP722602.1). The confirmed DNA was subsequently used as the positive control in all real-time PCR runs.

Each run included a positive control (*E. moshkovskii* reference DNA), species-specific negative controls (*E. histolytica* and *E. dispar* DNA), and a no-template control; however, the probe design provided high specificity, minimising the likelihood of cross-reactivity. To assess the potential diagnostic impact of sequence variability, *18S rRNA* sequences obtained from clinical isolates were compared in silico with the primer and probe binding regions described by Hamzah et al. [27] using Primer-BLAST (NCBI).

### Amplicon purification and Sanger sequencing

Amplified products were purified using the QIAquick PCR Purification Kit (QIAGEN, USA) according to the manufacturer’s protocol. DNA was eluted in 20 µL of elution buffer and sequenced bidirectionally at the Department of Mycology, PGIMER, employing the same primers used in the PCR assay. Chromatograms were manually analysed using FinchTv software, and consensus sequences were generated by aligning forward and reverse reads on MEGA 11 software. High-quality sequences were verified for base-calling accuracy and subsequently deposited in the NCBI GenBank database (PP059641.1, PP059638.1, PP059636.1, PP059634.1, PP059635.1, PP059622.1, PP059623.1, PP059619.1, PP059614.1, OR879976.1, OR879974.1, OR879972.1).

### Phylogenetic analysis

Consensus *18S rRNA* sequences from clinical isolates were aligned with reference *E. moshkovskii* sequences retrieved from GenBank using MUSCLE alignment implemented in MEGA 11 [25, 26]. Sequence identity was verified by BLASTN (basic local alignment search tool nucleotide) analysis. Twelve clinical isolates with high-quality chromatograms were retained for analysis; five sequences with ambiguous or poor-quality reads were excluded. The dataset included 12 clinical isolates from this study, along with six animal-derived, six human-derived, and six environmental *E. moshkovskii* reference sequences. Human-derived sequences (H1–H6) correspond to accession numbers KP722602.1, OM791622.1, OP537199.1, OP537196.1, ON965450.1, and KP722601.1; animal-derived sequences (A1–A6) correspond to OP453350.1, OP453352.1, MZ357997.1, MZ357991.1, MZ357989.1, and MN536492.1; environmental sequences (E1–E5) correspond to MN536496.1, KJ870232.1, MN536498.1, MN536497.1, and MN536494.1

Phylogenetic trees were reconstructed using the maximum likelihood (ML) method in MEGA 11, applying the Tamura–Nei substitution model. Tree robustness was evaluated through 1,000 bootstrap replicates. Rate variation among sites was modelled using a gamma distribution with invariant sites (G + I). Partial deletion with a 95% site coverage cutoff was applied to handle gaps, and the Nearest-Neighbour Interchange (NNI) method was used for heuristic search. The initial tree was generated using the NJ/BioNJ algorithm. Clades were interpreted based on isolate origin (clinical, animal, or environmental).

Although the amplified fragment was short (~ 128 bp), it encompassed species-informative polymorphisms sufficient for subclade discrimination, as previously demonstrated by Hamzah et al. [27] and subsequent comparative studies.

### Mutation identification and genetic variability

To identify genetic polymorphisms, consensus sequences from clinical isolates were aligned with the *E. moshkovskii* reference *18S rRNA* sequence (GenBank accession KP722602.1) using Clustal Omega. Alignments were manually curated in MEGA 11 to confirm true nucleotide substitutions and to exclude artefacts due to ambiguous bases or alignment mismatches. Single-nucleotide polymorphisms (SNPs) and deletions were catalogued relative to the reference sequence, and their distribution patterns were examined to infer possible evolutionary divergence.

### Statistical analysis

Demographic and clinical data were compiled in Microsoft Excel and analysed using *SPSS* version 16.0 (IBM, USA). Descriptive statistics summarised baseline characteristics and infection prevalence. The proportion of *E. moshkovskii*-positive cases was expressed as percentages with 95% confidence intervals (CIs). Differences in infection rates between age and sex groups were assessed using Fisher’s exact test or Chi-square test, as appropriate. A *p*-value < 0.05 was considered statistically significant.

## Results

### Microscopic and molecular detection

Among 300 hospitalised patients with acute diarrhoea, *Entamoeba* cysts or trophozoites were detected microscopically in 30 specimens (10.0%; 95% confidence interval [CI] 6.9–14.0%). However, morphological similarities among *Entamoeba* species precluded reliable species differentiation (Supplementary Fig. 1). The staining was performed using a modified trichrome protocol standardised in our laboratory. The clinical data of the patients infected with *E. moshkovskii* infection are given in Table 1.

**Table 1:** Clinical and demographic characteristics of patients infected with *E. moshkovskii* infection (*n* = 17/300)

Clinical conditions	Number of individuals infected
IBS	02 (0.66%)
Ulcerative colitis	02 (0.66%)
Crohn’s disease	01 (0.33%)
Pancreatitis	02 (0.66%)
Transplant patient (pre/post)	04 (1.33%)
Age (in years)	
< 18 years	07 (2.33%)
19-50 years	06 (2%)
> 50 years	04 (1.33%)
Sex (M/F)	
Male	11 (3.66%)
Female	06 (2%)

Molecular detection identified *Entamoeba moshkovskii* in 17 of 300 patients (5.7%; 95% CI 3.3–8.9%). Notably, three of these PCR-positive samples were negative by microscopy, underscoring the higher sensitivity of the molecular assay. Representative amplification plots and corresponding cycle threshold (Ct) values from the probe-based real-time PCR assay are provided in Supplementary Figure S2 and Supplementary Table S1. No other parasites (i.e. co-infection with *E. histolytica/ E. dispar/ Giardia/ Cryptosporidium*) were detected by both microscopy and molecular assay among these 17 samples.

The occurrence was higher among male patients (3.7%; 95% CI 1.8–6.5%) than female patients (2.0%; 95% CI 0.7–4.3%), though the difference was not statistically significant (*p* = 0.42). Age-stratified prevalence showed the highest positivity among individuals younger than 18 years (2.3%), followed by those aged 19–50 years (2.0%) and those older than 50 years (1.3%), with no significant variation among groups (*p* = 0.67).

No mixed *Entamoeba* infections were identified among *E. moshkovskii*–positive samples, and these findings collectively indicate that infection distribution in this cohort was not significantly influenced by age or sex.

### Sequencing and genetic confirmation

Bidirectional sequencing of the *18S rRNA* gene yielded 12 high-quality *E. moshkovskii* sequences. BLASTN analysis confirmed close genetic similarity with published *E. moshkovskii* references, showing 99–100% sequence identity. Several isolates were identical to prototype strains, while others exhibited one to three nucleotide variations. All validated sequences were submitted to the NCBI GenBank database under unique accession numbers (PP059641.1, PP059638.1, PP059636.1, PP059634.1, PP059635.1, PP059622.1, PP059623.1, PP059619.1, PP059614.1, OR879976.1, OR879974.1, OR879972.1).

### Mutation analysis

Alignment of clinical sequences with the *E. moshkovskii* reference strain (GenBank accession KP722602.1) revealed a consistent nucleotide substitution in isolates MOSHKO-10, MOSHKO-11, and MOSHKO-12. At position 1655, the reference thymine (T) was replaced by the IUPAC code “R” (adenine/guanine), representing a base transition. No substitutions were detected in isolate MOSHKO-4 relative to the reference sequence. Sporadic, ambiguous base calls observed in a few isolates were not reproducible and were interpreted as artefactual variations.

### Evaluation of primer and probe binding regions

Comparative in silico analysis of the 18S rRNA sequences obtained from clinical samples against the published *E. moshkovskii* primer and probe binding regions demonstrated complete conservation across all high-quality sequences. Although a few isolates showed ambiguous bases adjacent to—but not overlapping—the primer or probe binding sites, these variations did not alter predicted hybridisation efficiency. These findings confirm that the existing real-time PCR assay remains highly reliable for detecting *E. moshkovskii* in the local clinical population.

### Phylogenetic analysis

**Fig. 1:**
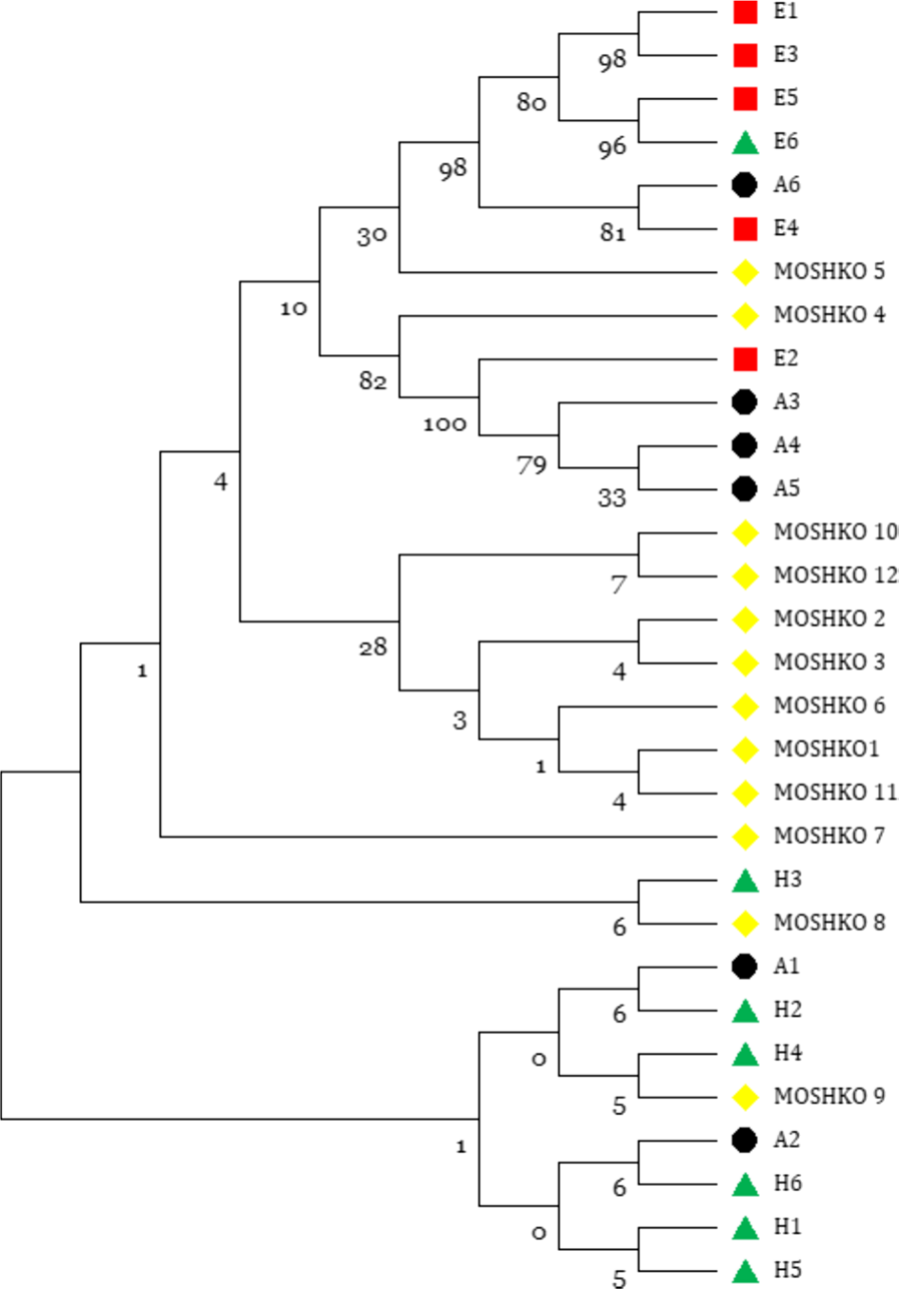
Maximum-likelihood phylogenetic tree showing evolutionary relationships among clinical, animal, human, and environmental isolates of *E. moshkovskii* based on *18S rRNA* gene sequences. Bootstrap values are indicated at branch nodes

Phylogenetic reconstruction based on the *18S rRNA* gene resolved three major clusters corresponding to human, animal, and environmental lineages (Fig. 1). The majority of clinical isolates (MOSHKO-1 to MOSHKO-3 and MOSHKO-5 to MOSHKO-12) grouped within a single, moderately supported cluster (bootstrap 66–73%), indicating high genetic relatedness among these isolates. Within this cluster, isolates MOSHKO-6 and MOSHKO-7 formed a distinct subclade (bootstrap ≈82%), and MOSHKO-1 was closely associated with MOSHKO-10 (bootstrap ≈67%).

Isolates MOSHKO-8 and MOSHKO-9 occupied intermediate positions between the clinical and human reference sequences, whereas MOSHKO-4 diverged from all other clinical isolates, aligning more closely with environmental and animal references (E2, A3–A5). Reference sequences segregated by origin: human-derived (bootstrap 81–83%), animal-derived (81–89%), and environmental (80–83%) clades were distinctly separated. The phylogenetic proximity of certain clinical isolates, particularly MOSHKO-4 and MOSHKO-5, to non-human references suggests possible overlap between human and environmental or zoonotic transmission routes (Table 2) (Fig. 1).

**Table 2:** Phylogenetic clustering of *Entamoeba moshkovskii* isolates based on *18S rRNA* sequences

Sample(s)/isolate(s)	Clustered with	Clade source	Bootstrap support (%)	Interpretation
MOSHKO-1–3, 5–7, 10–12	Main clinical cluster (monophyletic)	Clinical	66–73	Clonal cluster with modest divergence
MOSHKO-6, 7	Subclade within clinical cluster	Clinical	~ 82	Well-supported subclade
MOSHKO-1, 10	Subclade within clinical cluster	Clinical	~ 67	Close pair within main cluster
MOSHKO-8, 9	Adjacent to H2–H4, A2	Human + animal	68–73	Intermediate, possible cross-species link
MOSHKO-4	E2, A3–A5	Environmental + animal	79–100	Divergent lineage, environmental/animal linkage
MOSHKO-5	E1–E5, A6	Environmental + animal	96–98	Strongly supported mixed-source clade
H1–H6	Human references	Human	81–83	Distinct human clade
A1–A6	Animal references	Animal	81–89	Distinct animal clade
E1–E6	Environmental references	Environmental	80–83	Distinct environmental clade

## Discussion

This study provides the first sequence-confirmed evidence of *Entamoeba moshkovskii* infection among hospitalised patients with acute diarrhoea in Northern India and characterises its genetic diversity using the 18S rRNA gene. The molecular prevalence (5.7%) is comparable with reports from Bangladesh, eastern India, and Malaysia [27, 28], reinforcing that *E. moshkovskii* circulates widely in tropical regions with poor sanitation and water contamination. Its exclusive detection among symptomatic inpatients supports potential clinical relevance in this cohort, although the cross-sectional design does not permit causal inference. Further case–control or longitudinal studies will be essential to determine whether *E. moshkovskii* acts as a true enteropathogenic or as an opportunistic commensal under specific host conditions.

Phylogenetic reconstruction based on partial 18S rRNA sequences delineated three well-defined clusters corresponding to human, animal, and environmental origins. Most clinical sequences were grouped within a human-associated clade, demonstrating high relatedness among circulating strains, whereas MOSHKO-4 and MOSHKO-5 showed close affinity with animal and environmental reference sequences. Such topology suggests potential cross-ecological transmission and supports evidence of overlapping *E. moshkovskii* genotypes recovered from humans, pigs, and wastewater samples [28]. The detection of a reproducible single-nucleotide substitution T → R at position 1655 (relative to the reference strain KP722602.1) in three isolates may represent early microevolutionary divergence within the regional population. Limited bootstrap support in this analysis primarily reflects the high conservation of the 18S rRNA locus rather than true genetic uniformity—a constraint repeatedly observed in previous *E. moshkovskii* phylogenies [29]. Broader multilocus or whole-genome approaches, incorporating variable loci such as KERP1, amoebapore C, and chitinase, could better resolve population structure and clarify whether certain genotypes are linked to host adaptation or enhanced virulence [30].

From a diagnostic perspective, complete conservation across primer–probe binding regions of the Hamzah et al. [27] assay confirms its suitability for this setting. Nevertheless, the occurrence of ambiguous bases adjacent to these sites highlights the importance of continual local sequence validation to prevent target drop-out in molecular assays [31]. Periodic in silico evaluation and adoption of multiplex or multi-target PCR designs may further safeguard diagnostic accuracy in genetically diverse populations. Incorporating *E. moshkovskii* into standard molecular panels for diarrhoeal pathogens would help re-estimate the true burden of amoebic infections that may be misclassified as *E. histolytica* or *E. dispar* by microscopy alone. Such integration aligns with the One Health paradigm, recognising environmental and zoonotic reservoirs as contributors to human infection.

Beyond prevalence estimation, these molecular findings carry epidemiological and ecological implications. The coexistence of human-adapted and environmentally affiliated genotypes within the same clinical setting suggests that exposure likely occurs through contaminated water or food chains rather than direct person-to-person transmission. The detection of *E. moshkovskii* in treated wastewater and agricultural reuse systems in previous studies underscores its environmental resilience and potential for waterborne spread [28]. Strengthening wastewater monitoring and environmental surveillance could therefore complement clinical diagnostics and inform control strategies.

Despite its strengths, this study has several limitations. Genetic inference relied on a short 18S fragment that, while adequate for species identification, offers limited power for intraspecific resolution; bootstrap values should thus be interpreted cautiously. Only 12 of 17 PCR-positive samples produced high-quality bidirectional sequences, which might have underestimated sequence diversity. Although stool microscopy was performed, the absence of targeted bacterial culture or viral antigen testing restricts interpretation of causality and co-infection dynamics. The hospital-based design and focus on symptomatic patients further limit generalisability to community settings.

Nevertheless, the study has several merits. It was a prospective study with standardised sample collection, a validated probe-based real-time PCR for detection, and bidirectional Sanger sequencing for species confirmation of this elusive and neglected, yet epidemiologically important, protozoan. Inclusion of human, animal, and environmental reference sequences in phylogenetic reconstruction provided a broader ecological context, while deposition of all confirmed sequences in GenBank ensures transparency and reproducibility. The integration of clinical data with molecular characterisation strengthens confidence in the epidemiological interpretation and establishes a reference baseline for future comparative studies from Northern India.

Collectively, these results extend the molecular epidemiology of *E. moshkovskii* to an unreported region of India and highlight its genetic heterogeneity, environmental linkage, and diagnostic relevance. Continued surveillance using multilocus or genome-wide typing and inclusion of asymptomatic controls will be essential to elucidate transmission networks and define their precise role in human disease.

## Conclusion and public health significance

This study provides the first sequence-confirmed evidence of *Entamoeba moshkovskii* infection in patients with acute diarrhoea from Northern India. Phylogenetic clustering revealed distinct human, animal, and environmental lineages, suggesting overlapping transmission routes and shared ecological reservoirs. Although its pathogenic potential remains uncertain, the consistent detection of *E. moshkovskii* in symptomatic individuals and the observed genetic heterogeneity highlight its clinical and epidemiological importance.

Future research should employ multilocus or whole-genome approaches to resolve strain-level diversity and identify virulence or host-adaptation markers. Integration of One Health-based surveillance—encompassing human, animal, and environmental components—will be vital to delineate transmission dynamics and assess its broader public health relevance.

## Supplementary Information

**Additional file 1.**

## Data Availability

All nucleotide sequences generated and analysed during the current study are available in the NCBI GenBank repository under the submitted accession numbers [accession numbers will be provided upon acceptance].

## Abbreviations:

BLAST: Basic Local Alignment Search Tool
CI: Confidence interval
IEC: Institutional Ethics Committee
ML: Maximum likelihood
MLST: Multilocus sequence typing
NCBI: National Centre for Biotechnology Information
NJ: Neighbour-joining
PCR: Polymerase chain reaction
rRNA: Ribosomal ribonucleic acid
SNP: Single-nucleotide polymorphism
