# Supplementary material for: Emerging genetic diversity and molecular epidemiology of Entamoeba moshkovskii among patients with acute diarrhoea in Northern India
Source: Trop Med Health. 2026 Jan 3;54:3. doi: 10.1186/s41182-025-00876-5 (PMC12766943; doi:10.1186/s41182-025-00876-5)
Supplement: Supplementary file 1 — Additional file 1. [file 41182_2025_876_MOESM1_ESM.docx]

**Supplementary Figure S1**. Microscopic visualisation of *Entamoeba* species from stool samples. The left panel shows mature cysts with distinct nuclei and chromatoid bodies, while the right panel depicts trophozoites with ingested erythrocytes, characteristic of *Entamoeba* morphology (trichrome stain, 100× magnification).


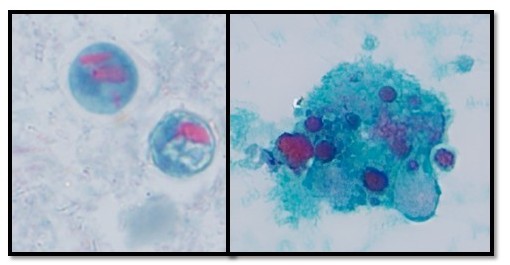


**Supplementary Figure S2.** Representative amplification plot from the probe-based real-time PCR assay targeting the 18S rRNA gene of *E. moshkovskii*. Distinct amplification is observed for the positive control (*E. moshkovskii* *PC*) and seven clinical isolates (*E. moshkovskii* 1–7), while *E. histolytica*, *E. dispar*, and the no-template control (NC) show no amplification, confirming assay specificity.


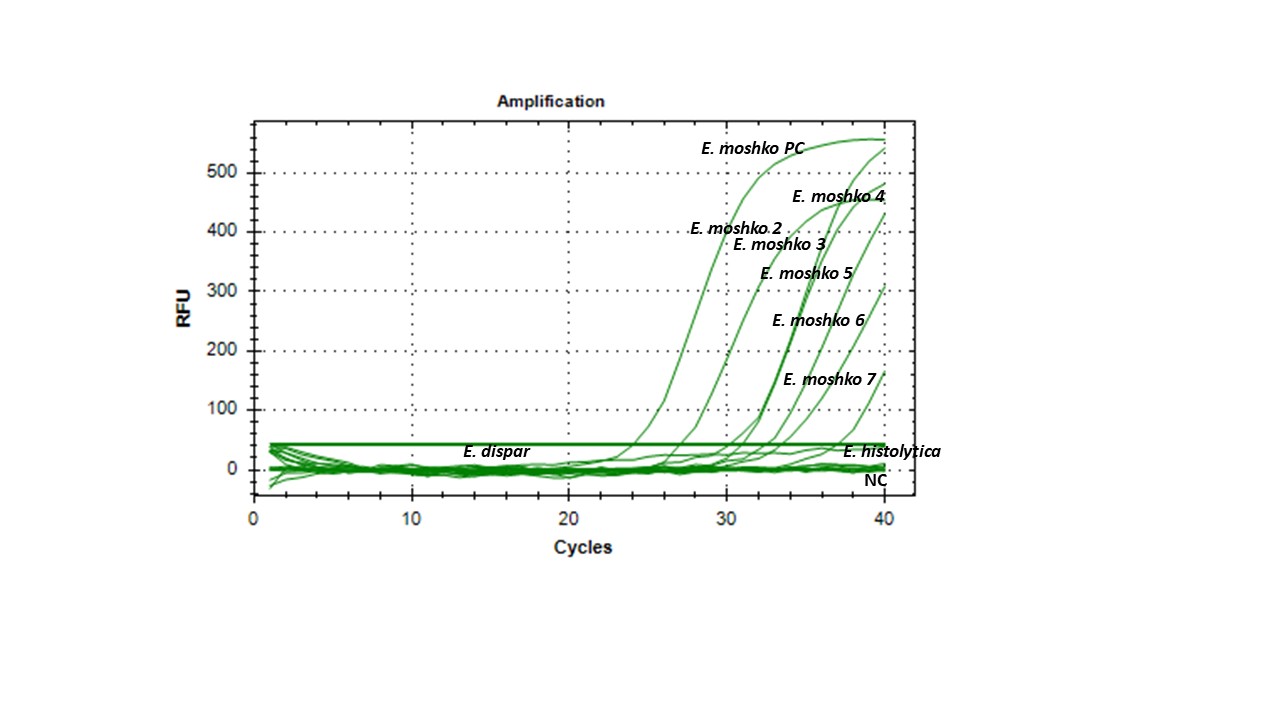


**Supplementary Table S1.** CT values of probe-based real-time PCR assay targeting the 18S rRNA gene of *E. moshkovskii* in clinical samples and controls. “–” indicates no amplification.

| **Sample ID** | **Ct Value** | **Result** | **Control Type** |
| --- | --- | --- | --- |
| *E. moshkovskii* ***1*** | 27.4 | Positive | Clinical |
| *E. moshkovskii* ***2*** | 29.1 | Positive | Clinical |
| *E. moshkovskii* ***3*** | 30.5 | Positive | Clinical |
| *E. moshkovskii* ***4*** | 28.3 | Positive | Clinical |
| *E. moshkovskii* ***5*** | 32.0 | Positive | Clinical |
| *E. moshkovskii* 6 | 33.7 | Positive | Clinical |
| *E. moshkovskii* ***7*** | 31.9 | Positive | Clinical |
| *E. moshkovskii* **PC** | 23.5 | Positive | Positive Control |
| ***E. histolytica*** | – | Negative | Negative Control |
| ***E. dispar*** | – | Negative | Negative Control |
| **NC** | – | Negative | No-template Control |
